# Xylem vessel anatomy and hydraulic function scale in concert along the tip-to-base axis of an angiosperm tree

**DOI:** 10.1093/aobpla/plaf072

**Published:** 2025-12-10

**Authors:** Swetlana Kreinert, Lucian Kaack, Luciano Pereira, Marcela T Miranda, Stefan Mäck, Jonas Schuler, Steven Jansen

**Affiliations:** Institute of Botany, Ulm University, Albert-Einstein-Allee 11, Ulm 89081, Germany; Institute of Botany, Ulm University, Albert-Einstein-Allee 11, Ulm 89081, Germany; Institute of Botany, Ulm University, Albert-Einstein-Allee 11, Ulm 89081, Germany; Laboratory of Crop Physiology (LCroP), Department of Plant Biology, Institute of Biology, University of Campinas (UNICAMP), Rua Monteiro Lobato 255, 13083-862, Campinas, SP, Brazil; Institute of Botany, Ulm University, Albert-Einstein-Allee 11, Ulm 89081, Germany; Institute of Botany, Ulm University, Albert-Einstein-Allee 11, Ulm 89081, Germany; Institute of Botany, Ulm University, Albert-Einstein-Allee 11, Ulm 89081, Germany; Form & Function

**Keywords:** angiosperm xylem, *Fagus sylvatica* L., vessel scaling, vessel diameter, vessel length, vessel lumen conductivity, vessel end-wall conductivity, vessel widening, vessel lengthening, hydraulic resistivity

## Abstract

Vessel scaling from tip to base in angiosperms has largely been studied based on vessel diameter. Here, we test if vessel anatomy and transport efficiency in a *Fagus sylvatica* L. sapling show axial scaling by maintaining a largely proportional ratio of lumen to end-wall resistivity to sap flow with tree height. Vessel diameter (*D*) of more than 50 000 vessels was measured based on wood sections, while mean vessel length (*L_V_*) was measured semi-automatically with a Pneumatron for 58 stem segments. Based on tip-to-base variation in *D* and *L_V_*, we estimated vessel lumen conductivity (*K_H_*) at the individual vessel level. We also estimated end-wall conductivity (*K_W_*) based on Darcy’s law, integrating pit membrane thickness (*T*_PM_) with scaling of *D* and total inter-vessel pit membrane area (*A_P_*) across the sapling. Axial variation in *K_W_* was evaluated against end-wall pressure difference (ΔP). In addition to a tip-to-base increase in *D*, we found an increase in *L_V_* and *A_P_*, illustrating basipetal vessel lengthening. These patterns were associated with proportional changes in *K_W_* and *K_H_*, which followed a 1:1 relationship with distance to the tip, each contributing to ∼ 50% of the whole-tree conductivity/resistivity. Our findings suggest that vessel dimensions and hydraulic functionality show axial scaling in angiosperm trees, suggesting that anatomy corresponds to the adjustment of hydraulic functionality with plant height. Proportional adjustment of *K_W_* and *K_H_* highlights the key role of vessel dimensions and inter-vessel pits in regulating transport efficiency and safety, potentially maintaining constant resistance per unit leaf area with height growth.

## Teaser

Trees rely on tube-like vessels to transport water, yet how these vessels adjust along a plant's height is not fully understood. We studied a Fagus sylvatica sapling to test whether vessel anatomy and hydraulic conductivity scale consistently from the tip to the base. By measuring vessel diameters, lengths, and pit membrane traits, we estimated both lumen conductivity and end-wall conductivity for individual vessels. We found that vessels become wider, longer, and have larger pit membrane areas toward the base, and that these anatomical shifts produce matched increases in both lumen and end-wall conductivity. This 1:1 scaling suggests that trees maintain a stable water transport efficiency along their height.

## Introduction

Woody angiosperms can transport water through unicellular tracheids and multicellular vessels, which consist of a series of vessel elements interconnected by perforation plates (Evert 2006). The dimension and spatial arrangement of vessels play a major role in sustaining long-distance water transport in trees (Zimmermann and Tomlinson 1966). While vessel diameter has been given considerable attention as vessel widening in studies on tip-to-base scaling (Anfodillo et al. 2013, Olson and Rosell 2013, Olson et al. 2014, Rosell et al. 2017, Hacke et al. 2022, Jacobsen and Pratt 2023, Petit et al. 2023, Petit 2024), there is rather limited evidence for vessel length scaling, and how vessel anatomy and sap transport capacity vary along the vertical axis of angiosperm trees (Comstock and Sperry 2000, Lens et al. 2022).

Vessel diameters in xylem of woody plants show a considerable variation in size, ranging from 10 µm to > 300 µm, which indicates their functional significance for diverse water transport strategies (Olson and Rosell 2013, Rosell et al. 2017, Fajardo et al. 2020, Soriano et al. 2020). Although vessel diameters are relatively easy to measure in cross-sections using light microscopy, narrow vessels cannot be easily distinguished from tracheids in transverse sections (Scholz et al. 2013). Multiple studies show conduit widening from tip to base across a wide range of terrestrial vascular plants, approximating a power law, with pronounced changes in diameter at the tips, and a slow rate of widening at the base of the stems (West et al. 1999, Anfodillo et al. 2013, Olson et al. 2014, 2021, Rosell et al. 2017, Echeverría et al. 2019, Williams et al. 2019, Koçillari et al. 2021). This vessel diameter scaling with plant height has been functionally explained by the need to maintain a similar transport efficiency and hydraulic resistivity as a tree grows taller (Petit et al. 2008, Anfodillo et al. 2013). Indeed, hydraulic resistivity (i.e. the resistance normalized to the length of a stem or root, or to the entire xylem pathway length of a tree) is the reciprocal of conductivity, with both scaling to the fourth power of the vessel diameter according to the Hagen–Poiseuille law. Determining hydraulic resistivity and conductivity has been largely hampered by technical shortcomings in measuring vessel length, which may not be fully overcome by empirical approaches. If tip-to-base conduit widening compensates for the increase in hydraulic path length due to tree height growth, then leaf-specific hydraulic conductance should remain constant regardless of height. This requires that the sources of resistance in the xylem, which do not include the lumen diameter only, scale proportionally to maintain a balance in total tree resistance for a given height. Anatomical features that impose additional resistance, such as inter-vessel pit membranes, must be compensated for by other structural adjustments, such as greater vessel widening and/or lengthening. This provides a framework to evaluate whether the various elements of xylem architecture evolve or develop in coordinated ways to sustain constant hydraulic efficiency across the vertical growth of trees. Without this proportional consistency in hydraulic vessel properties, tree height would be strongly limited by its hydraulic properties.

The resistance to water flow is not only due to the diameter (i.e. the hydraulic lumen resistance) but also arises from the interconnected vessel areas (also called end walls), and therefore the mean vessel length (*L_V_*) (Wheeler et al. 2005, Cai and Tyree 2014). Vessel length can vary significantly, ranging from less than 1 mm to several metres across species, even within a single plant (Zimmermann et al. 1981, Ewers et al. 1990). Nowadays, *L_V_* can be acquired in a straightforward and rather fast approach using a Pneumatron device (Pereira et al. 2020, Peng et al. 2022). The estimation of the vessel length distribution and *L_V_* are important factors when evaluating hydraulic efficiency due to the direct effect of vessel length on conductivity (Hacke et al. 2006, Loepfe et al. 2007). Ewers et al. (1990) found a linear correlation between max vessel diameter and max vessel length, while Hacke et al. (2006) found an interspecific logarithmic relationship between *L_V_* and mean vessel diameter. Although a scaling between vessel length and vessel diameter is likely, there is a clear need to study this scaling in detail, both across species and within a single tree (Pan et al. 2015, Liu et al. 2018).

The natural selection for organisms that aim to minimize the hydrodynamic resistance of their vascular system has been described by West et al. (1997, 1999) in the fractal-like model known as the West, Brown, and Enquist model (hereafter the WBE). A fractal is a complex geometrical shape that exhibits self-similarity and scaling behaviour. An example of a fractal in two-dimensional geometry is the Pythagorean tree, which resembles branching structures found in nature. This tree-like structure is used in mathematical modelling, computer sciences, and simulations to study natural systems, particularly how branching optimizes space and resource distribution (i.e. structure of binary hierarchies—file systems or phylogenetic trees) (Pourahmadazar et al. 2011, Beck et al. 2014). The WBE model suggests that all vascular plants (and many other organisms) follow a scaling rule to minimize flow resistance and optimize size-invariant flow by adjusting the size of each observed fractal, which is represented by the scaling of vascular bundles across the height of a plant. One of the main constraints of this model is the assumption that the vascular network is composed of tubes of identical length (West et al. 1999, Kozłowski and Konarzewski 2004). The diameter of the tubes, however, is allowed to vary and therefore contributes to the tapering between each fractal element (West et al. 1999). The general allometric scaling law, as suggested by WBE, results in a fixed scaling exponent of vessel diameters and consequently, fluid flow per tube, with this scaling exponent being multiples of one-fourth for vascular plants (West et al. 1997, 1999).

Since vessel length and vessel diameter are related to lumen resistivity, and potentially total inter-vessel pit membrane area (*A_P_*) and pit membrane thickness (*T*_PM_), pit characteristics may determine flow between vessels and therefore end-wall resistivity. Mature pit membranes are composed of cellulose microfibril aggregates and represent fibrous porous media with a species-specific thickness between ∼ 160 and 1000 nm (Kaack et al. 2021). Pits function as valves that control the liquid–gas interfaces between an embolized and sap-filled vessel and therefore play an important role in embolism propagation (Kaack et al. 2021, Isasa et al. 2023). Similar to *L_V_* measurements, multiple steps in sample preparation and data analysis are involved in estimations of *T*_PM_ and *A_P_* (Wheeler et al. 2005, Jansen et al. 2009).

Zimmermann and Brown (1971) showed that vessel length and diameter play an important role in hydraulic conductivity measurements. Further studies suggested that vessel end walls account for ∼ 56% of the total vessel hydraulic resistance (Sperry et al. 2005, Hacke et al. 2006), limiting water transport efficiency in a largely one-by-one relationship with vessel lumen resistance. Therefore, the total xylem resistance and permeability are determined by the dimensions and arrangement of vessels, and by pit membrane thickness and porosity (i.e. the pore volume fraction) of pit membranes. Furthermore, *A_P_* plays a role in transport efficiency (Wheeler et al. 2005, Hacke et al. 2006, Pereira et al. 2023). The ratio between lumen and end-wall conductivity demonstrates the functional significance of vessel length and diameter as well as inter-vessel connectivity. Increasing vessel length results in lower total resistivity (i.e. the vessel resistance normalized to the vessel length), because there are fewer end walls to cross for a given vessel length, as well as a lower end-wall resistivity (Sperry et al. 2005, Wheeler et al. 2005).

Traditionally, hydraulic conductivity has been measured experimentally using xylem segments, which are generally longer than the maximum vessel length (Scholz et al. 2013, Balaz et al. 2016). It is known, however, that hydraulic measurements can be prone to various artefacts and inconsistencies, making stable measurements and integration of vessel length distribution difficult to achieve (Kolb and Sperry 1999, De Baerdemaeker et al. 2019). While theoretical conductivity estimations may overcome this problem, the challenge here is to account for inter-vessel end walls. Yet, it seems logical to consider the vessel length as a functional unit to estimate conductivity. Since the axial hydraulic conductivity follows a vessel widening pattern, it can be expected that this tip-to-base widening is associated with an increased vessel length. Besides vessel dimensions, the inter-vessel end-walls are proposed to be the major anatomical driver for a potential, convex relationship between hydraulic safety and efficiency between species, with *T*_PM_ and *A_P_* being the main determinants (Pereira et al. 2023). According to Darcy’s law, the pressure difference across bordered pits in a vessel end-wall scales proportionally with pit membrane thickness (*T*_PM_) and is inversely related to the total inter-vessel pit membrane area (*A_P_*). A convex trade-off or exponential relationship between safety and efficiency was recently described for the end-wall pressure difference, which represents a proxy for safety, while vessel end-wall conductivity is an indicator of hydraulic efficiency (Pereira et al. 2023). Overall, modelling hydraulic conductivity based on detailed anatomical parameters provides a valid approach with considerable advantages as compared to experimental measurements of hydraulic conductivity and resistivity.

Here, we aim to investigate the allometric relationship between vessel length (*L_V_*), vessel diameter (*D*), and total inter-vessel pit membrane area (*A_P_*) at a whole-tree level from the apical meristems of the main stem and branches to the stem base. Specifically, we test whether there is vertical variation along a stem in vessel diameter (*D*) and vessel length (*L_V_*), and whether or not an axial pattern affects *A_P_*. We hypothesize that *D*, *L_V_*, and *A_P_* scale proportionally from tip to base, and that the ratio of lumen to end-wall conductivity across a tree is constant. Therefore, axial variation in these vessel dimensions within a single tree may follow a proportional logarithmic relationship. If this hypothesis could be supported, there would be no major hydraulic limitation to growth in height by the xylem tissue. Consequently, the absolute conductivity would change from tip to base, but the ratio of lumen to end-wall conductivity would be constant, following a 1:1 relationship throughout the whole tree. These findings have considerable consequences for safe and efficient water transport in xylem tissue (Loepfe et al. 2007). We ignore in this study the potential resistance offered by hydraulic segmentation (also called compartmentalization and sectoriality; Zimmermann and Brown 1971, Zanne et al. 2006, Levionnois et al. 2020). Although segmentation can be common as local hydraulic bottlenecks at branch bifurcations, or stem-petiole and stem-branch transitions (André et al. 1999, Guan et al. 2021), its overall contribution to resistance at the whole-plant level is assumed to be relatively low.

While this study is the first empirical test for angiosperms, prior theoretical work has anticipated our main hypothesis. Koçillari et al. (2021) and Olson et al. (2021) both proposed that anatomical resistances, such as pit membranes, internal sculpture, and vessel length, must scale in concert with conduit widening to maintain functional consistency of sap flow during height growth. Although roots and leaves were not included in this study, we suggest that the observed trends may apply to the entire xylem pathway. Moreover, empirical tests confirmed scaling patterns in tracheid-baring conifers, where the total bordered pit area per tracheid scales isometrically with lumen size (Lazzarin et al. 2016, Zambonini et al. 2024), but no such tests have been conducted for *A_P_* in vessel-bearing angiosperms, making this study novel.

## Material and methods

### Plant material and sampling

An entire 2.75 m tall sapling of *Fagus sylvatica* L. was sampled in April 2021 at the botanical garden of Ulm University, Germany. Each lateral branch of interest was harvested successively, starting at the base of the main stem, and used to measure the mean vessel length (*L_V_*). In total, we sampled 20 branches, including the main stem (sampling scheme Supplementary Fig. S8). For each branch, we collected its length (distance to the branch tip). Each branch was divided into two to eight segments, depending on its total length. The length of each segment, which varied from 5 to 43 cm, was selected to have no or few open vessels in each segment, with longer segments for the more basal, thicker branches, and shorter sections for apical, thinner segments. The length of open vessels corresponded to vessels with maximum length, which had been estimated based on trials. Since vessel length distribution is strongly short-skewed, the occurrence of a few open vessels in some segments was unproblematic and did not affect the overall estimation of the mean vessel length (Cohen et al. 2003, Cai and Tyree 2014, Pereira et al. 2020).

We also collected samples of each segment used for *L_V_* measurements for wood anatomical measurements. The wood samples were preserved in 75% ethanol and stored in a fridge until sectioning.

### Vessel length distribution

Vessel length distribution analysis was performed using a Pneumatron device (Pereira et al. 2020, Peng et al. 2022). To determine the mean vessel length, each segment (*n* = 58) was connected at its base to the device *via* a silicone tube and sealed with clamps to prevent leakage. The gas conductivity was measured successively after shortening the segment by cutting. The shortening of the segment led to an exponential increase in gas conductivity when more and more cut-open vessels were obtained, especially when the segments became very short. The vessel probability distribution function (*P_x_*), as well as the mean vessel length, was calculated according to Cohen et al. (2003) (1).


(1) $$P_{x}=x{\;\lambda }_{v}^{2}\;\exp(\lambda_{v}x)$$

Where *x* was the stem length and λ*_v_* was the slope of the gas conductivity vs. *x*. The mean vessel length was −2/λ*_v_* (Cohen et al. 2003).

### Light microscopy and scanning electron microscopy

Transverse sections from the base of each segment were prepared with a sliding microtome (Microtome—GSL 1, Switzerland), and had a thickness of 20–30 µm. The sections were stained with 0.05% toluidine blue. Images were made using a stereo microscope (Axio Zoom.V16, Zeiss, Jena, Germany) and analysed using ImageJ. For each segment, the outermost tree rings were analysed. Between 333 and 1000 vessels per segment were measured. For young branches with a diameter of < 3 mm, all vessels were analysed, except for the protoxylem ones. For all 58 samples, a total of 59 000 vessel diameters was obtained.

The remaining wood sample was used for scanning electron microscopy (SEM). The wood samples were cut longitudinally and fixed on aluminium sample holders. The samples were sputtered (Balzers Union, FL—9496 Principality of Liechtenstein) with gold for 2 min, resulting in a coating of about 30 nm in thickness. Images of at least three to five individual pit-fields per segment were obtained using a desktop SEM (Phenom XL, Thermo Fisher Scientific, Germany). The images were analysed using ImageJ (Schindelin et al. 2012). Within each pit-field, a minimum of 10 pits were measured. A total of 3000 pits were measured for 58 samples.

We followed Scholz et al. (2013) to determine various anatomical traits. The equivalent circle diameter (*D*) (2) was calculated using the vessel lumen area (*A*).


(2) $$D=\;\sqrt{\frac{4A}{\pi }}$$

Furthermore, the inter-vessel contact fraction (*F_C_*) was determined as the ratio of the sum of the inter-vessel contact perimeter (*L*_VW_) to the sum of the total conduit perimeter (*P_V_*) (3).


(3) $$Fc=\;\frac{\sum L_{\mathrm{vw}}}{\sum P_{v}}$$

The inter-vessel pit fraction (*F_P_*) was determined (4) as the product of Fc and the ratio of the inter-vessel pit area per total inter-vessel wall (i.e. the pit field fraction, *F*_PF_).


(4) $$Fp=F_{C}\times F_{\mathrm{PF}}$$

The inter-vessel pit membrane area [mm^2^] (*A_P_*) was determined for each sample (5) using *F_C_*, *F_P_*, as well as the mean equivalent circle diameter (*D*) and the mean vessel length (*L_V_*) determined for each sample individually (Wheeler et al. 2005).


(5) $$A_{P}=F_{C}\;\times F_{P}\;\times D\;\times L_{V}$$

### Calculation of hydraulic parameters

Our approach to estimate the lumen hydraulic conductivity (*K_H_*) in units of m^3^ Pa^−1^ s^−1^ (6) was adapted from Tyree and Zimmermann (2002) by including vessel length. This approach differed from a more frequent normalization by considering the length of a given segment that is used for hydraulic measurements. Here, *K_H_* was calculated for each segment using the mean vessel diameter (*D*), mean vessel length (*L_V_*), as well as dynamic water viscosity (μ = 0.001002^−^ Pa s).


(6) $$K_{H}=\;\frac{\pi \;\times \;D^{4}}{128\;\times \;\mu \;\times \;L_{V}}$$

Hydraulic resistance is inversely proportional to hydraulic conductance, which was based on the Hagen–Poiseuille equation (Sperry et al. 2005, Wheeler et al. 2005, Hacke et al. 2006, Pittermann et al. 2006).

To calculate the end-wall conductivity (*K_W_*) (7) as well as the end-wall pressure difference (ΔP) (8) (Pereira et al. 2023), we estimated the pit membrane permeability (*k* = 1.00^−13^ m^2^). This estimation was based on the empirical evidence that lumen vs. end-wall resistivity follows a 1:1 line, with end-wall resistivity forming approximately 50% of total resistivity and the normalization to sample length (Sperry et al. 2005). Our calculations were normalized to the mean vessel length measured. Pit membrane thickness (*T*_PM_ = 234 nm) measurements were taken from published data (Kaack et al. 2021), as well as constant end-wall pressure difference = 1, and axial variation in pit membrane thickness was assumed to be negligible based on earlier evidence (Klepsch et al. 2018, Kotowska et al. 2020).


(7) $$K_{W}=\;\frac{k\;\times \;A_{P}\times \;1\;}{\mu \;\times \;T_{PM}}$$

End-wall conductivity was calculated for each segment with constant pit membrane thickness, but *A_P_* values were estimated for each individual segment. End-wall resistivity was inversely proportional to end-wall conductivity.

We assumed flux (*Q* = 5 × 10^−13^ m^3^ s^−1^) across end walls to be constant based on Pereira et al. (2023) to calculate the pressure difference (ΔP, Pa) (8) across end walls.


(8) $$\Delta P=\;\frac{\eta \;\times \;T_{\mathrm{PM}}\;\times Q}{k\;\times \;A_{P}}$$

### Relating classes of vessel length to corresponding vessel diameter classes

To show the relationship between vessel diameter and vessel length (Supplementary Fig. S5), the data were first filtered and sorted by vessel diameter. We followed the assumption that a large vessel diameter was associated with a long mean vessel length.

Secondly, a sequence of simulated vessel groups was created. Each vessel group or class contained 10 vessels. The sequence of vessel length sizes within a group was calculated using the mean vessel length measured. For this grouping, we took one-third and three times the mean vessel length as the upper and lower boundaries, assuming that the mean vessel length and the maximum vessel length follow a linear relationship.

Then, the relative vessel diameters were attributed to each vessel length group by filtering the relative diameters that were larger than a given vessel length group, and smaller than the successive, longer vessel length group. This resulted in classes of possible vessel lengths and their associated vessel diameter. The frequency of each diameter class was stored as the number of vessels (*D*). The frequency of 0 was omitted, as we did not have a class of 0 vessel groups.

### Data analysis

The data were processed using R programming in the RStudio environment (R Core Team 2020). The linear models were estimated and plotted using the libraries ‘GPUBR’, ‘SEGMENTED’, and ‘GGPLOT2’ (Muggeo 2008, Wickham 2016). We used the piecewise linear regression to demonstrate the relationship between measured vessel diameter and estimated vessel length (Supplementary Fig. S5). This analysis allowed the separation of data into distinct intervals. Each interval was fitted separately, which was particularly useful when the relationship between the independent variable (measured vessel diameter) and the dependent variable (estimated vessel length) changed at a specific point, referred to as the breakpoint.

We assessed model assumptions by testing residual normality (Lilliefors test), homoscedasticity (Breusch–Pagan test), and independence (Durbin–Watson test). Additionally, diagnostic plots (residuals vs. fitted, Q–Q plot, scale–location, and residuals vs. leverage) were used to visually evaluate model fit and potential violations. We estimated the trend between the variables using the non-parametric Theil–Sen estimator, calculating the median of all pairwise slopes with intercept derived from the median residuals. Kendall’s tau correlation test was applied to assess monotonic relationships, and a 95% confidence interval for the slopes was obtained from the distribution of the pairwise slopes. Furthermore, we applied log-transformed linear regression models to demonstrate relationships between variables, extracting slopes to evaluate the scaling relationship of the given traits. The samples were divided into three equal-sized categories: segment diameters with a small, intermediate and large diameter, since the segment diameter was directly related to the segment position in the tree as well as the age of the branch or segment. The deviation from the 1:1 line in Fig. 5 was calculated using root mean square deviation (RMSD) analysis.

## Results

### Anatomical scaling from tree tip to base

The mean vessel diameter of the whole sapling was 22.55 µm, of the main stem 20.3 µm, of the first-order branches 16.05 µm, and of the second-order branches 14.52 µm. The mean vessel length measured of the stem and all branches was 5.7 cm, of the main stem 8.3 cm, of the first-order branch 5.2 cm, and 5.3 cm of the second-order branches.

**Figure 1:**
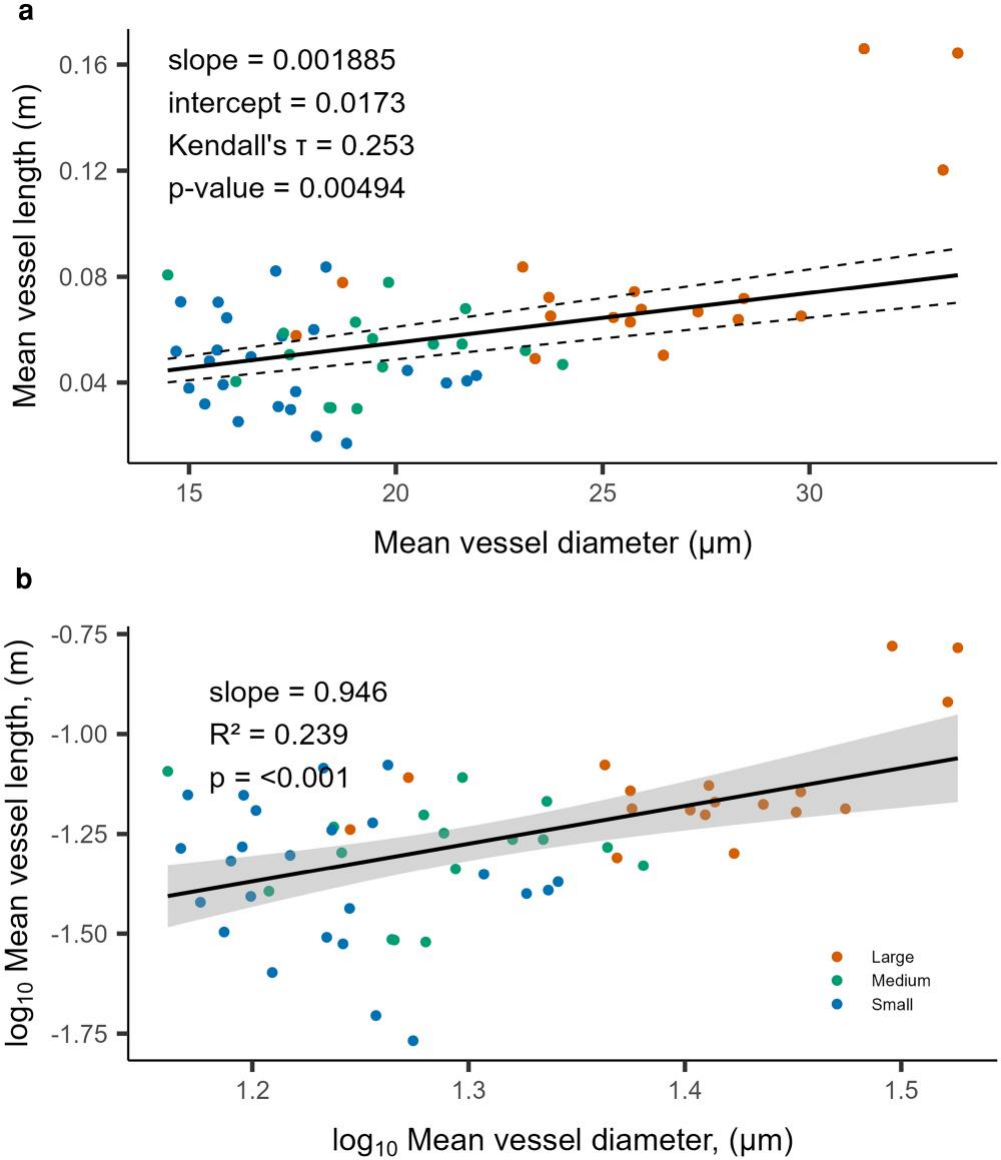
Mean vessel length (*L_V_*) plotted against mean vessel diameter (*D*) for 58 wood segments from a *Fagus sylvatica* L. sapling (a) and log transformed data in (b). Each dot represents values of a segment from the main stem, and all branches. The length of this segment was selected to have no or few open vessels (i.e. vessels with a length longer than the segment). The black line indicates the non-parametric linear regression between *L_V_* and *D* with 95% CI (dashed lines).

We identified a positive linear correlation between the mean vessel diameter and the mean vessel length, as well as a linear relationship using log-transformed data (Fig. 1). The mean diameter increased with length, with a slope of 0.0173 for the non-parametric approach and 0.946 for the logarithmic approach, with each point in Fig. 1 representing an individual segment of the entire tree.

Furthermore, we found a linear relationship between both the mean diameter and mean vessel length with the distance of each sampled segment to the tip of the main stem (Supplementary Figs S1 and S2).

A similar positive and linear relationship was found between the grouped mean vessel diameter and the grouped, estimated vessel length (see Methods for details). However, we found higher slopes for the segments of the stem and the branches of the first order, and lower slopes in the segments of the second order (Supplementary Fig. S5). On the other hand, the intercept was similar among the stem and branch orders. This similarity suggested that the deviation from ideal linear proportionality did not differ between the main stem and branches, and that an equivalent scaling trend occurred.

### Hydraulic scaling at the whole-tree level from tip to base

**Figure 2:**
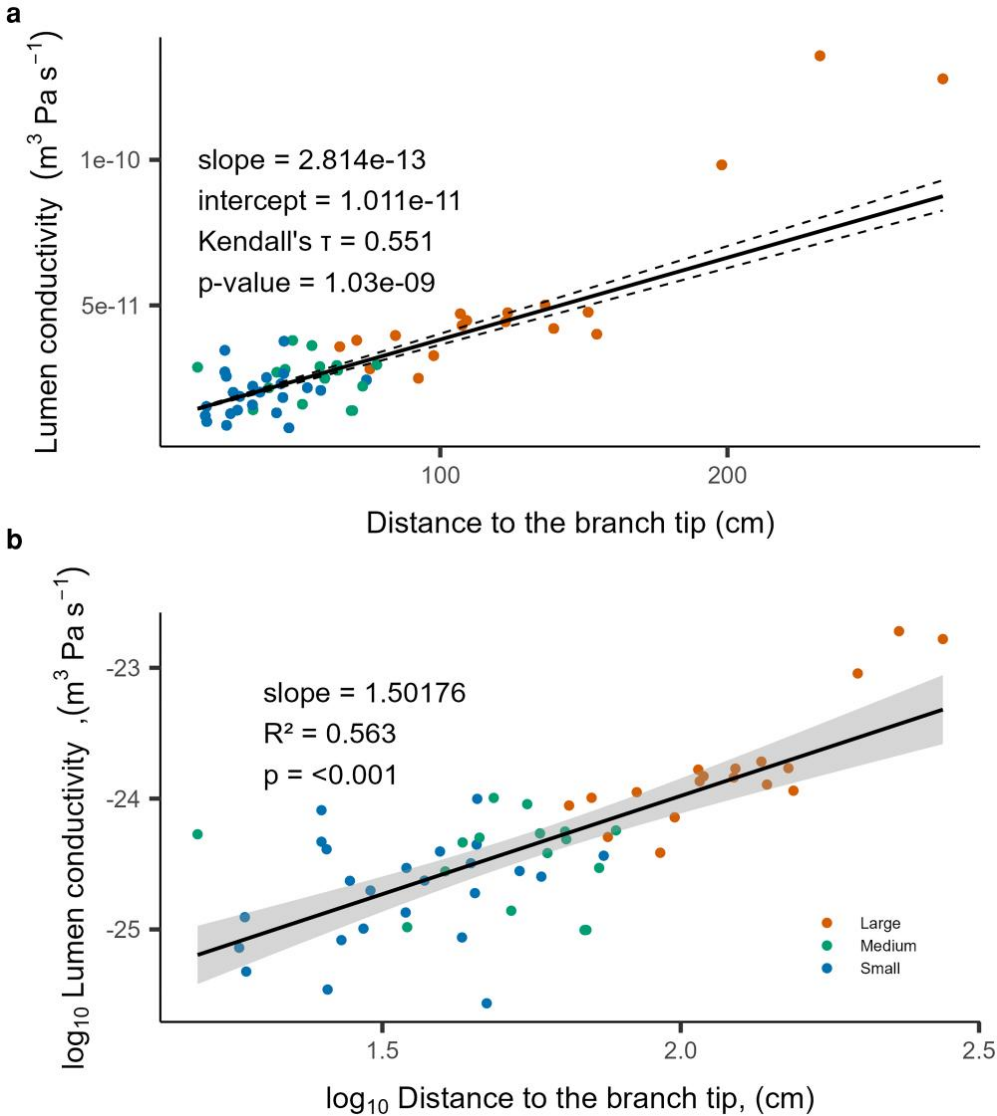
Relationship between the vessel lumen conductivity (*K_H_*) and segment position in the tree separated by segment base diameter (large, medium, and small). The black line shows the non-parametric linear regression and Kendall tau correlation as well as the 95% CI (dashed lines) (a) and log-transformation of the pooled data in (b). Each dot represents a segment of the sapling studied.

Vessel lumen conductivity (*K_H_*) increased from the apical tips to the base, with the steepest increase occurring within the main stem (Fig. 2). As lumen conductivity was a function of both vessel length and diameter, it reflected the relationship between mean vessel diameter and mean vessel length. Therefore, vessels that were wide and long exhibited the highest lumen conductivity and lowest resistivity.

**Figure 3:**
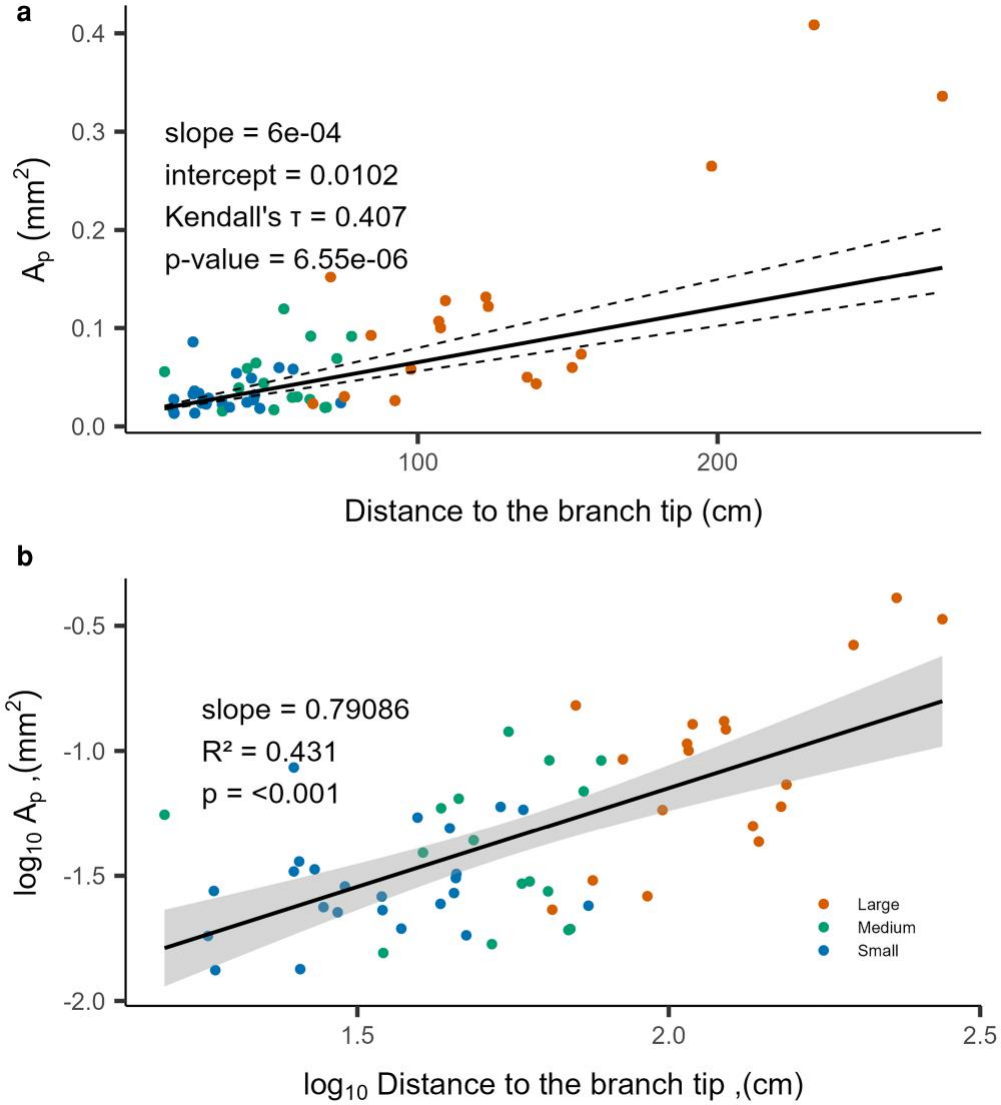
Relationship between the average inter-vessel pit membrane area per vessel (A*_P_*; mm^2^) and segment position in the tree, with non-logarithmic (a) and log-transformed data (b). The black line indicates the non-parametric linear regression of *A_P_* and the distance from the segment base to the tip of each branch (*n* = 58), and the dashed lines represent the 95% CI. Each dot represents a segment of the sapling studied.

We identified an increase in *A_P_* throughout the tree height, with highest *A_P_* values for segments closest to the base of the tree, and lowest to the stem tip (Fig. 3). Furthermore, we found a weak relationship between axial height and inter-vessel contact fraction (*F_C_*), and between axial height and pit field fraction (*F*_PF_), indicating low or no scaling for these parameters (Supplementary Figs S3 and S4).

**Figure 4:**
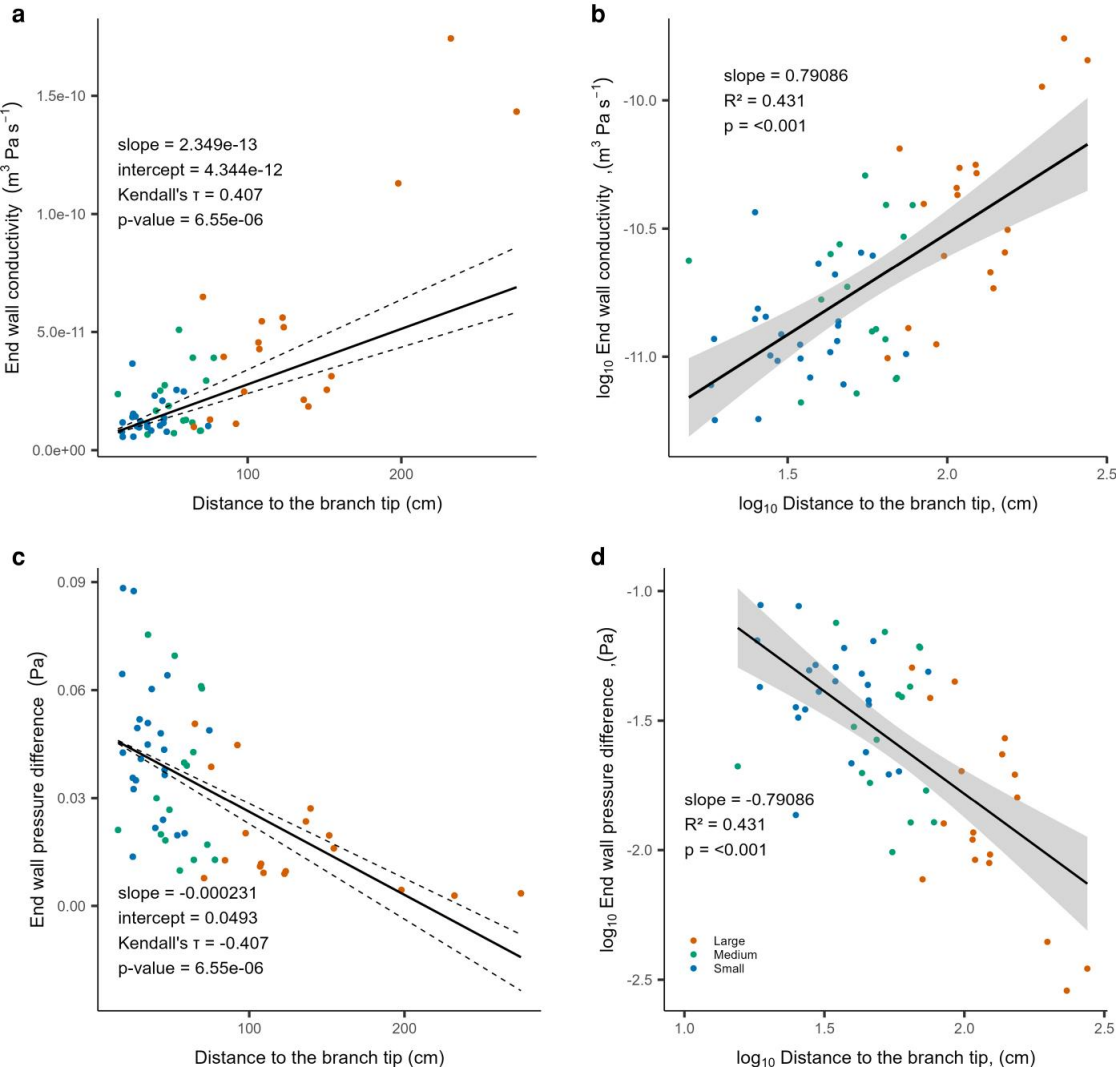
Relationship between the vessel end-wall conductivity (*K_W_*) and the end-wall pressure difference (ΔP) with segment position in the tree with non-logarithmic (a, c) and with log-transformed data in (b) and (d). ΔP (Pa) behaves directly inverse to end-wall conductivity. The highest pressure difference and lowest end-wall conductivity occur at the tips of the branches and the tip of the stem.

Narrow vessels with short mean vessel lengths were found at the tip of the main stem and branches (small segment diameters in blue), showing very low lumen conductivity. A similar pattern was also observed for vessel end-wall conductivity. End-wall conductivity scaled with the distance to the branch tip as well as in dependency of segment diameter, and consequently with vessel diameter and vessel length (Fig. 4). The wider the diameter and the longer the vessels, the higher the end-wall conductivity.

End-wall pressure difference followed an inversely proportional relationship to end-wall conductivity, and therefore increased towards the tip of the main stem and the tips of the branches (small segment diameter in blue) (Fig. 4).

**Figure 5:**
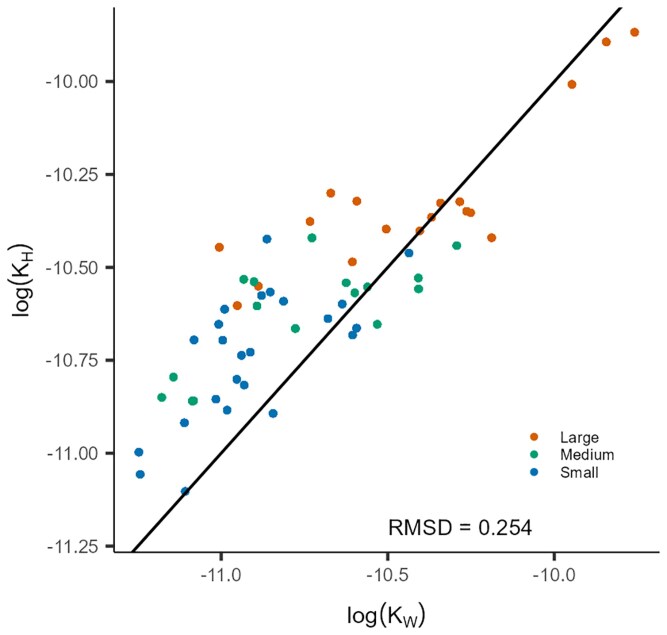
Relationship between the log_10_ transformed vessel end wall (*K_W_*) and vessel lumen conductivity (*K_H_*), which were normalized to the average vessel length for each segment. The black line represents the 1:1 line. The deviation from the 1:1 line was tested using RMSD analysis (RMSD = 0.254) (*n* = 58).

Vessel end-wall conductivity, with vessel length included in its estimation, was close to a 1:1 proportionality (log_10_-transformed data) with vessel lumen conductivity. This scaling applied both to the branch orders and the main trunk of the sapling (Fig. 5). Conductivities were calculated for each segment, thus capturing variation in vessel diameter as well as changes in mean vessel length throughout the segments measured. Consequently, both the vessel end-wall conductivity and vessel lumen conductivity accounted for ∼ 50% of the total resistivity of the whole tree.

The deviation from the 1:1 line (RMSD = 0.254) suggested a diminishing return effect, meaning that increases in *K_W_* lead to proportionally smaller increases in *K_H_*. Considering that the pit membrane permeability (*k*) was unknown, our best estimation of its value should result in a proportional relationship between *K_W_* and *K_H_*, which would be in line with empirical evidence that *K_W_* represents ∼ 50% of the total conductivity (Sperry et al. 2005). Therefore, we tested different values of *k*, and used the best fit to the 1:1 line, which was found for a value of *k* = 1.00^−13 ^m^2^. We also tried *k* values up to 1.00^−17^ m^2^, which gave the strongest discrepancy between the y-axis (*K_H_*) and the x-axis (*K_W_*).

### No hydraulic limitation at the tree level

**Figure 6:**
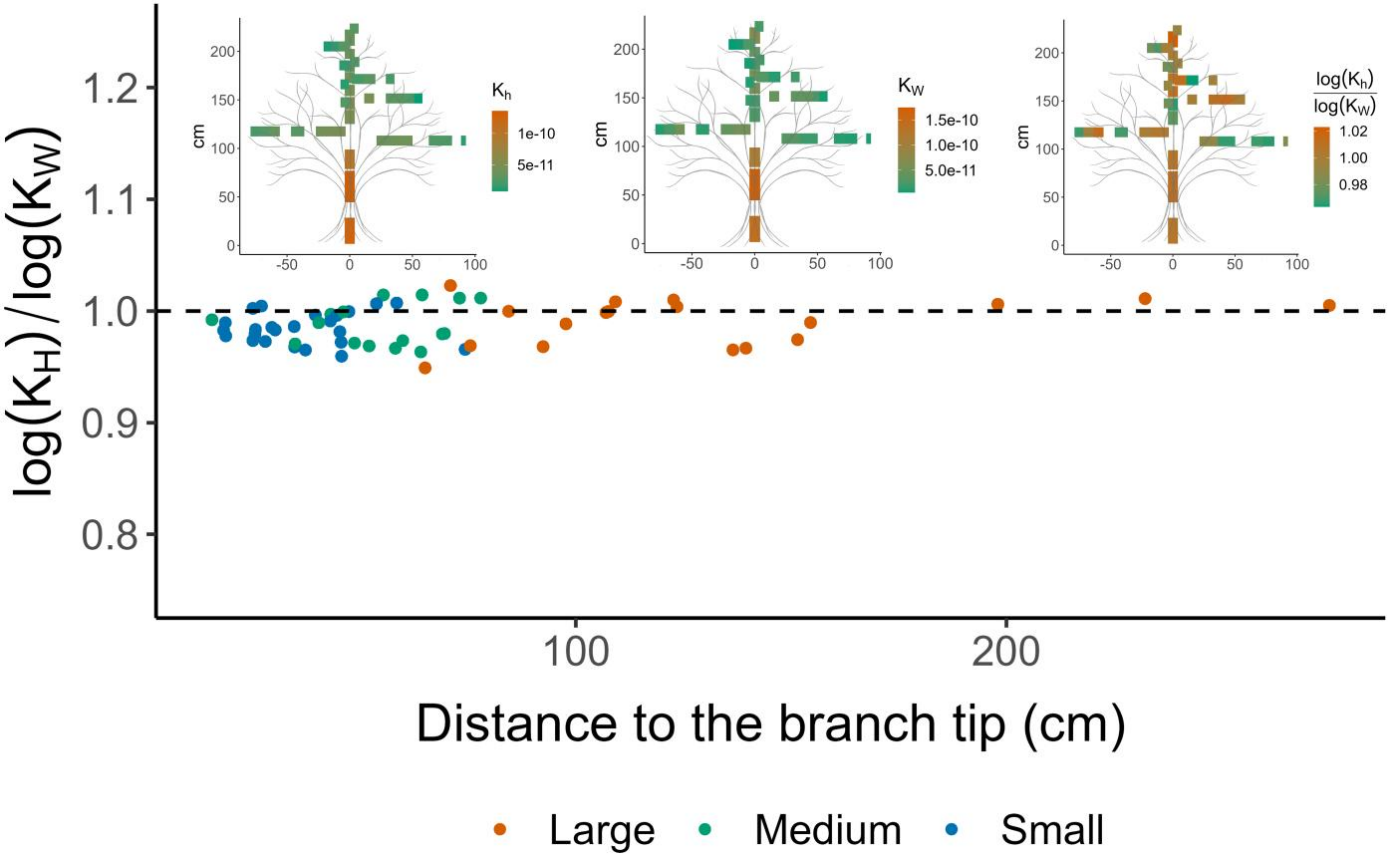
The ratio of lumen conductivity to end-wall conductivity (*K_H_*/*K_W_*) in relation to the segment distance to the tip. Each dot represents a segment. The insert graphs show the position of the segments within the tree, and the color gradient represents the distribution of *K_H_*, as well as *K_W_* and the *K_H_*/*K_W_* ratio. In total, most segments show an equal contribution of *K_H_* to *K_W_* resulting in a ratio of ∼ 1.

To explore how vessel lumen and vessel end-wall conductivity scale within the tree, we calculated their ratio. Values of this ratio ranged from a minimum of 0.95 to a maximum of 1.02 across the whole tree. These values covered samples from the main trunk to the first and second branch orders, and were in line with our hypothesis that no hydraulic limitation would occur within the xylem irrespective of its height (Fig. 6).

The ratio of vessel lumen to vessel end-wall conductivity showed little deviation from 1, indicating a proportional relationship between these conductivities regardless of the position of the segment used for the calculation.

## Discussion

### Scaling of vessel dimensions within a tree optimize hydraulic efficiency

Based on vessel lumen and vessel end-wall conductivities (Figs 2 and 4), xylem hydraulic adjustment to height was found from the tip to the base. Despite changes in quantitative xylem anatomical dimensions, the safety and efficiency proxies (ΔP and *K_H_/K_W_*) are adjusted proportionally, resulting in no major constriction for water transport along the xylem hydraulic pathway from the stem base to the tip of branches (Fig. 6; Supplementary Fig. S6; Ewers et al. 1990, Hacke et al. 2006, Sperry et al. 2006, Cai et al. 2010). Although we did not study roots and xylem of leaves, our data suggest that a plant’s entire xylem transport system is structured in such a way that it minimizes hydrodynamic resistance, as predicted by the WBE model (West et al. 1997, 1999). Moreover, a novel finding of our study is clear evidence of vessel lengthening (Supplementary Fig. S2) along with vessel widening (Olson and Rosell 2013, Rosell et al. 2017, Soriano et al. 2020, Olson et al. 2021) also appeared to be associated with an increasing trend in total pit membrane area per vessel (*A_P_*) from tip-to-base (Fig. 3), which is reported here for the first time.

The scaling patterns observed also show functional effects on hydraulic conductivity, and its non-linear trade-off with safety (Supplementary Fig. S6). We identified no axial variation in the ratio of vessel end-wall conductivity to vessel hydraulic conductivity, both for the main stem and branches (Fig. 6). As the WBE model suggested, a constant ratio or proportionality is important for energy conservation and flow optimization (West et al. 1997, 1999, Brown et al. 2005).

While the WBE model provides a useful framework, its predictions for hydraulic efficiency are based on idealized assumptions, including both ideal fractal scaling and the simplified representation of conduits as perfect, continuous capillaries without internal resistances like perforation plates, inter-vessel pit membranes, or vessel length effects (Haberlandt 1914, West et al. 1999, Petit and Anfodillo 2009, Balaz et al. 2016, Koçillari et al. 2021). Real plants deviate from these idealized fractal geometries and perfect conduit assumptions, which results in a convex trade-off between maximizing vessel area for water transport and minimizing transport distances to reduce energy expenditure in building pathways or to reduce resistivity. While the exact nature of simplifying assumptions of the WBE model can be debated, the powerful prediction remains that all sources of resistance must scale in harmony to achieve efficiency. Although selection favours narrow conduits at the distal end to enable efficient regulation and exchange, maintaining sufficient flow over longer distances requires progressive tip-to-base vessel widening. This anatomical adjustment, comparable with a fractal-like arrangement of branches or segments (conceptual illustration of a wood segment interpreted as a fractal unit, Supplementary Fig. S7), reduces hydraulic resistance and enhances overall conductance. Optimization of the vessel surface area is also achieved by varying *A_P_* within an individual plant, and *T*_PM_ interspecifically, while vessel dimensions (*L_V_* and *D*) affect transport efficiency, and allow for a functional adjustment of the vessel area to maintain the total hydraulic conductance (Price and Enquist 2006).

### Safety scaling within a tree

Hydraulic scaling by adjustment of vessel dimensions likely explains the absence of any major hydraulic constraint within the aboveground xylem of the sapling studied. An axial scaling pattern supports a gradual transmission of the water potential from the leaves to the roots, while ensuring efficient water supply to the crown. This scaling pattern accommodates pressure changes across end walls as well as environmental challenges, such as drought susceptibility of distal branches (Johnson et al. 2016, Lechthaler et al. 2020). The axial water potential gradient is inversely proportional to vessel lumen and vessel end-wall conductivity (Sperry et al. 2005).

By calculating pressure differences based on the mean vessel length as a reference unit, our data show hydraulic adjustment based on variation in vessel diameter, vessel length, and total pit area across a tree, while pit membrane thickness varies mainly between species (Pereira et al. 2023). However, pit membrane thickness has also been hypothesized to decrease with increasing plant height in support of the ‘ultra-widening permeability’ hypothesis (Lens et al. 2011, Isasa et al. 2023, Anfodillo and Olson 2024). End-wall pressure differences for basal stem segments appear to be steeper than those for the tip of the main stem and branches (Fig. 4; Supplementary Fig. S6), which is in line with acropetal declines in vessel lumen and vessel end-wall conductivity, and reflected in the anatomical scaling of vessels (Supplementary Fig. S5). The main stem exhibits a higher scaling slope, indicating a stronger scaling effect than the branches, which could be explained by the adjustment to higher total conductivity and high water transport efficiency near the stem base. This observation can also be attributed to the deviation of the basal stem segments from the 1:1 line (Fig. 5), meaning that the total hydraulic conductivity is driven by vessel lumen conductivity, although few end walls only need to be crossed when vessels are wide and long, which means that minor changes in end-wall conductivity lead to proportionally small changes in vessel lumen conductivity. The same can be observed with basal stem segments showing a higher ratio than the 1:1 line in the *K_H_/K_W_* ratio (Fig. 6). A ratio higher than 1 indicates that vessel lumen conductivity increases faster than end-wall conductivity within the studied segment, which could be hydraulically favourable for basal tree stems. The opposite pattern can be observed for segments of higher order or tips of the branches, where vessels are narrower and shorter. Clearly, further analyses of how variation in tree architecture, including both single-stem and multi-stem trees, affects hydraulic scaling patterns would be an interesting topic for further research.

A hydraulic tree map (Fig. 6) or analysis of scaling patterns of xylem illustrates the relationship between vessel end-wall and vessel lumen conductivity, vessel diameter, and vessel length (Figs 2 and 4 and Supplementary Figs S1 and S2; Tyree and Ewers 1991, Cruiziat et al. 2002). The observation that tree architecture follows a scaling pattern is consistent with Leonardo da Vinci’s early hypothesis that the transverse sectional area of a tree trunk equals the sum of its branches’ transverse sectional areas, possibly the first-documented observation of scaling in trees and ever since described in the context of tip-to-base vessel widening along the tree trunk (Tyree and Zimmermann 2002, Olson and Rosell 2013, Rosell et al. 2017, Olson et al. 2021). While there are various interpretations of Da Vinci’s rule in literature—ranging from the total area of the mother branch apex vs. the sum of daughter branch basal areas, to considerations of sapwood or only conductive tissue—and trees depart from Da Vinci’s rule in their external stem dimensions (Anfodillo and Olson 2024), the principle of maintaining constant conductance to the leaves across branches remains hydraulically valid. Indeed, maintaining consistent conductance per unit leaf area is crucial for efficient water transport, and this physiological requirement is often more consistent with a Widened Pipe Model (Koçillari et al. 2021). Nevertheless, Da Vinci’s early insight into the proportional relationships within tree architecture provided a foundational perspective for hydraulic tree mapping.

High end-wall pressure differences across end walls and embolism resistance are interspecifically associated with thicker pit membranes (*T*_PM_), and possibly low inter-vessel pit membrane area per vessel (*A_P_*) (Pereira et al. 2023). A convex trade-off between safety and efficiency within a tree may represent an adjustment to pressure changes along the height gradient by decreasing *A_P_* and increasing *T*_PM_ towards the branch and stem tip (Supplementary Fig. S6). Small changes in *T*_PM_ can have significant effects (Kaack et al. 2021). Although we assume *T*_PM_ to be constant within a tree, the available evidence suggests that pit membrane thickness may indeed slightly increase towards the apical tips (Klepsch et al. 2018, Kotowska et al. 2020, Guan et al. 2022, Levionnois et al. 2022). *T*_PM_ measurements can be challenging due to the time-consuming nature of its measurements, as well as potential shrinkage during the growing season (Schmid and Machado 1968, Sorek et al. 2021, Carmesin et al. 2023). Given these difficulties, combined with the fact that even small differences in thickness can drastically alter conductance, functionally significant variations might easily fall within the range of methodological error. This scenario, which could be compared to the ‘rare allele effect’ in population studies (Mouillot et al. 2013), where the most impactful variation is the hardest to detect, highlights the need for more detailed observations of *T*_PM_. Therefore, further work on this topic at the individual tree level would certainly be required.

Adjustment of *A_P_* would mean that hydraulic efficiency can either be facilitated or reduced, while hydraulic safety may only be affected by *A_P_* for a specific *T*_PM_ range (Kaack et al. 2021, Pereira et al. 2023). The potential adjustment and partial uncoupling of safety and efficiency reflect the scaling behaviour of the anatomical and hydraulic traits measured, allowing for efficient water transport while maintaining control over resistance to embolism across the height and thus pressure gradient. Although it seems reasonable to assume that the end-wall pressure difference is a proxy for safety, we lack methods to confirm that the embolism resistance of individual vessels increases with increasing end-wall pressure difference. While plant organs may show differences in embolism resistance based on the vulnerability segmentation hypothesis, there is no evidence for this hypothesis for a wide range of species (Klepsch et al. 2018, Levionnois et al. 2020, Peters and Choat 2024, Zambonini et al. 2024), which is in line with the functional interpretation of our findings, and the lack of a direct, mechanistic link between embolism resistance and vessel diameter (Lens et al. 2022, Isasa et al. 2023; Anfodillo and Olson 2024). Unfortunately, there is no standard method available to study embolism resistance of tree trunks (Bouche et al. 2016). On the other hand, there is a correlation between low *A_P_* and high embolism resistance based on the pit area hypothesis, which highlights the trade-off between safety and efficiency in xylem hydraulics at an interspecific level (Wheeler et al. 2005, Pereira et al. 2023). At the intraspecific level, segments of branches with low *A_P_* values and small vessel dimensions exhibit a relatively high end-wall pressure difference and could be on the safe end in the convex trade-off of safety and efficiency (Supplementary Fig. S6). The relationship between large *A_P_* and low embolism resistance of xylem was hypothesized based on the pit area hypothesis (Wheeler et al. 2005, Kaack et al. 2019) and should not be misinterpreted or mislabelled as the rare pit hypothesis. Clearly, the latter should be rejected because it is not compatible with the nature of pit membranes, especially their given thickness and multi-layered structure with several pore constrictions per pore pathway (Plavcová et al. 2013, Kaack et al. 2019, Carmesin et al. 2023, Zhang et al. 2024). Accordingly, the probability of embolism propagation increases with increasing *A_P_* for a given range of pit membrane thickness (Wheeler et al. 2005, Hacke et al. 2006). Furthermore, we found our results to align with predictions of ‘ultra-widening permeability’, suggesting that while tip-to-base vessel widening lessens the accumulation of hydraulic resistance with increasing pathlength, vessel widening may not entirely compensate for it (Anfodillo and Olson 2024). This indicates that additional factors, beyond conduit widening, likely contribute to the observed increase in permeability towards the base. These ‘ultra-widening’ mechanisms are hypothesized to be related to potential widening of vessel diameters in terminal branches with height growth (Zach et al. 2010, Olson et al. 2018), which would increase conductance throughout the plant (Echeverría et al. 2019), as well as other factors like inter-vessel pit membrane thickness or pit membrane area.

### Vessel diameter and vessel length follow a fractal-like scaling pattern

Our data are in line with previous studies that demonstrated a scaling between mean vessel diameter and mean vessel length, both intraspecifically (Cai et al. 2010) and interspecifically (Ewers et al. 1990, Hacke et al. 2006, Sperry et al. 2006), with narrow vessels being generally short, while wide vessels are rather long (Supplementary Fig. S5). Earlier reports also demonstrate logarithmic vessel widening across stem height, both interspecific or for an individual tree within the main stem, aligning with our finding of similar relationship between vessel diameter and tree height and distance from the stem tip (Supplementary Fig. S1; (Olson and Rosell 2013, Rosell et al. 2017, Olson et al. 2021). To further explore the dimensional relationship between vessel diameter and length (Supplementary Figs S2 and S5), we estimated vessel lengths for given vessel diameters based on the assumption that wide vessels tend to be long, as has previously been described (Hacke et al. 2006, Sperry et al. 2006, Jacobsen et al. 2012).

By applying different size classes of vessels, we observed that both short and narrow vessels, and long and wide ones, occur in basal segments of branches of higher order, but the overall scaling was that predominantly narrow and short vessels occur at the tips of branches and the tip of the main stem (Supplementary Fig. S5). This finding is consistent with the hypothesis that selection maintains constant leaf-specific conductance with height growth, favouring longer vessels when they are wider to optimize the scaling of resistance and conductance. This suggests a fractal-like organization of vessels with specific diameters and lengths within a tree or sampled segment (Supplementary Fig. S7), forming a structure that scales according to hydraulic demands. While the strict mathematical definition of ‘fractal’ in models like WBE refers to the dimensionality of space-filling networks rather than necessarily self-similar elements, the observed pattern of shorter, narrower vessels at the tip and longer, wider vessels towards the base plays a functional role in reducing hydraulic resistance, which is crucial for efficient water transport throughout the tree. The vessel connectivity explanation in Loepfe et al. (2007) further applies to the scaling patterns of vessel length and diameter, and to the occurrence of paired *L_V_* to *D* values. Short, wide vessels with low connectivity (low *A_P_*) are unlikely to occur, as they require a high supply to maintain, similar to a motorway with wide, short roads but few access points, which would remain mostly empty. The relatively low correlation between mean vessel length and diameter (Fig. 1) can be attributed to the method used for vessel length measurement in this study, as well as the assumption of random distribution of vessels.

It is crucial to understand the impact of vessel length on the calculation of hydraulic parameters. The estimation of vessel end-wall conductivity relies on the calculation of *T*_PM_ as well as *A_P_*, which gives equal mathematical weight to mean vessel length (*L_V_*) and mean vessel diameter (*D*) values. In contrast, lumen conductivity is more influenced by *D* than *L_V_* (Hagen–Poiseuille). For larger segments, *D* was measured using the outermost tree rings, while for smaller branches (1–3 mm), it was measured across the entire cross-section. Considering potential quantitative differences between juvenile and mature xylem, it is crucial to understand the impact of *L_V_* and *D* on hydraulic safety and efficiency of trees. Most studies use specific hydraulic conductivity (*K_s_*) as a measure of efficiency, which considers mean vessel diameter normalized to segment length, although strictly speaking, indices such as *K_s_* should be normalized by leaf area (Echeverría et al. 2019). However, *K_s_* primarily reflects the space use efficiency of wood tissues for sustaining hydraulic conductance, rather than hydraulic efficiency at the vessel level (Bittencourt et al. 2016). Our analysis incorporates measurements of *D*, *L_V_*, *A_P_*, and *T*_PM_, therefore resulting in *K_H_* and *K_W_* values with a more direct and precise estimation of hydraulic efficiency. These traits are also mechanistically linked to safety due to the critical role of multi-layered pit membranes in embolism resistance (Kaack et al. 2021, Pereira et al. 2023).

## Conclusion

We demonstrate that scaling of end-wall resistance to lumen resistance remains constant within the beech sapling studied. This finding can be explained by the scaling of xylem anatomical traits from the tip to the base, which ensures a proportional water transport efficiency and mitigates hydrodynamic resistance (in support of the WBE model), highlighting also an intra-individual convex trade-off between safety and efficiency. Furthermore, we demonstrate that hydraulic safety and efficiency within a tree is achieved through a dynamic interplay of vessel dimensions (*D* and *L_V_*) as well as *A_P_* and pit membrane thickness (*T*_PM_). By incorporating *D*, *L_V_*, *A_P_*, and *T*_PM_, we provide a mechanistic understanding of xylem water transport, supporting the importance of integrating vessel length in future studies. Further work would be needed to see how the distribution and frequency of tracheids as unicellular conduits affect scaling patterns and their functional significance in wood (Carlquist 1984), or how local sectoriality at nodes or transitions may affect axial scaling patterns, and whether pit membrane thickness scales across tree height accordingly.

## Supplementary Material

plaf072_Supplementary_Data

## Data Availability

Raw data and R code are available as Supporting information.

## References

[plaf072-B1] André JP, Catesson AM, Liberman M et al Characters and origin of vessels with heterogenous structure in leaf and flower abscission zones. Canadian Journal of Botany 1999;77:253–261. 10.1139/b98-213

[plaf072-B2] Anfodillo T, Olson ME. Stretched sapwood, ultra-widening permeability and ditching da Vinci: revising models of plant form and function. Ann Bot 2024;134:19–42. 10.1093/aob/mcae054 38634673 PMC11161570

[plaf072-B3] Anfodillo T, Petit G, Crivellaro A. Axial conduit widening in woody species: a still neglected anatomical pattern. IAWA J 2013;34:352–64. 10.1163/22941932-00000030

[plaf072-B4] Balaz M, Jupa R, Jansen S et al Partitioning of vessel resistivity in three liana species. Tree Physiol 2016;36:1498–507. 10.1093/treephys/tpw081 27609805

[plaf072-B5] Beck F., Burch M., Munz T. et al Generalized pythagoras trees for visualizing hierarchies. In Proceedings of the 5th International Conference on Information Visualization Theory and Applications - Vol. 1: IVAPP, (VISIGRAPP 2014). p.17-28. 10.5220/0004654500170028

[plaf072-B6] Bittencourt PRL, Pereira L, Oliveira RS. On xylem hydraulic efficiencies, wood space-use and the safety-efficiency tradeoff: comment on Gleason et al. (2016) weak tradeoff between xylem safety and xylem-specific hydraulic efficiency across the world’s woody plant species. New Phytol 2016;211:1152–5. 10.1111/nph.14044 27345844

[plaf072-B7] Bouche PS, Jansen S, Sabalera JC et al Low intra-tree variability in resistance to embolism in four Pinaceae species. Ann For Sci 2016;73:681–9. 10.1007/s13595-016-0553-6

[plaf072-B8] Brown JH, West GB, Enquist BJ. Yes, west, brown and enquist’s model of allometric scaling is both mathematically correct and biologically relevant. Functional Ecology 2005;19:735–8. 10.1111/j.1365-2435.2005.01022.x

[plaf072-B9] Cai J, Tyree MT. Measuring vessel length in vascular plants: can we divine the truth? History, theory, methods, and contrasting models. Trees 2014;28:643–55. 10.1007/s00468-014-0999-9

[plaf072-B10] Cai J, Zhang S, Tyree MT. A computational algorithm addressing how vessel length might depend on vessel diameter. Plant Cell Environ 2010;33:1234–8. 10.1111/j.1365-3040.2010.02142.x 20199614

[plaf072-B11] Carlquist S . Vessel grouping in dicotyledon wood: significance and relationship to imperforate tracheary elements. Aliso J Syst Floristic Bot 1984;10:505–25. 10.5642/aliso.19841004.03

[plaf072-B12] Carmesin CF, Port F, Böhringer S et al Ageing-induced shrinkage of intervessel pit membranes in xylem of *Clematis vitalba* modifies its mechanical properties as revealed by atomic force microscopy. Front Plant Sci 2023;14:1002711. 10.3389/fpls.2023.1002711 36755701 PMC9899931

[plaf072-B13] Cohen S, Bennink J, Tyree M. Air method measurements of apple vessel length distributions with improved apparatus and theory. J Exp Bot 2003;54:1889–97. 10.1093/jxb/erg202 12815034

[plaf072-B14] Comstock JP, Sperry JS. Theoretical considerations of optimal conduit length for water transport in vascular plants. New Phytol 2000;148:195–218. 10.1046/j.1469-8137.2000.00763.x

[plaf072-B15] Cruiziat P, Cochard H, Améglio T. Hydraulic architecture of trees: main concepts and results. Ann For Sci 2002;59:723–52. 10.1051/forest:2002060

[plaf072-B16] De Baerdemaeker Niels J.F, Keerthika Nirmani, Ranathunga Arachchige et al The stability enigma of hydraulic vulnerability curves: addressing the link between hydraulic conductivity and drought-induced embolism. Tree Physiology 2019;39. 10.1093/treephys/tpz078 31274162

[plaf072-B17] Echeverría A, Anfodillo T, Soriano D et al Constant theoretical conductance via changes in vessel diameter and number with height growth in Moringa oleifera. J Exp Bot 2019;70:5765–72. 10.1093/jxb/erz329 31328237 PMC6812708

[plaf072-B18] Evert RF . Esau’s Plant Anatomy: Meristems, Cells, and Tissues of the Plant Body: Their Structure, Function and Development, 3rd edn. Hoboken, New Jersey, Vereinigte Staaten: John Wiley Sons, Inc, 2006. https://books.google.de/books?hl=de&lr=&id=0DhEBA5xgbkC&oi=fnd&pg=PR7&dq=ray+evert&ots=r89q3iX7hl&sig=sv9K3Ho2LzQXiYSdxk9YLQ-R590#v=onepage&q=ray%20evert&f=false

[plaf072-B19] Ewers FW, Fisher JB, Chiu S-T. A survey of vessel dimensions in stems of tropical lianas and other growth forms. Oecologia 1990;84:544–52. 10.1007/BF00328172 28312972

[plaf072-B20] Fajardo A, Martínez-Pérez C, Cervantes-Alcayde MA et al Stem length, not climate, controls vessel diameter in two trees species across a sharp precipitation gradient. New Phytol 2020;225:2347–55. 10.1111/nph.16287 31657018

[plaf072-B21] Guan X, Pereira L, McAdam SAM et al No gas source, no problem: proximity to pre-existing embolism and segmentation affect embolism spreading in angiosperm xylem by gas diffusion. Plant Cell Environ 2021;44:1329–45. 10.1111/pce.14016 33529382

[plaf072-B22] Guan X, Werner J, Cao KF et al Stem and leaf xylem of angiosperm trees experiences minimal embolism in temperate forests during two consecutive summers with moderate drought. Plant Biol 2022;24:1208–23. 10.1111/plb.13384 34990084

[plaf072-B23] Haberlandt G . Physiological Plant Anatomy, 4th edn. London: MacMillan and Co. Limited St. Martins Street, 1914.

[plaf072-B24] Hacke UG, Jacobsen AL, Pratt RB. Vessel diameter and vulnerability to drought-induced embolism: within-tissue and across-species patterns and the issue of survivorship bias. IAWA J 2022;105:304–19. 10.1163/22941932-bja10107

[plaf072-B25] Hacke UG, Sperry JS, Wheeler JK et al Scaling of angiosperm xylem structure with safety and efficiency. Tree Physiol 2006;26:689–701. 10.1093/treephys/26.6.689 16510385

[plaf072-B26] Isasa E, Link RM, Jansen S et al Addressing controversies in the xylem embolism resistance–vessel diameter relationship. New Phytol 2023;238:283–96. 10.1111/nph.18731 36636783

[plaf072-B27] Jacobsen AL, Brandon Pratt R, Tobin MF et al A global analysis of xylem vessel length in woody plants. Am J Bot 2012;99:1583–91. 10.3732/ajb.1200140 22965850

[plaf072-B28] Jacobsen AL, Pratt BR. Vessel diameter polymorphism determines vulnerability-to-embolism curve shape. IAWA J 2023;6:1–15. 10.1163/22941932-bja10115

[plaf072-B29] Jansen S, Choat B, Pletsers A. Morphological variation of intervessel pit membranes and implications to xylem function in angiosperms. Am J Bot 2009;96:409–19. 10.3732/ajb.0800248 21628196

[plaf072-B30] Johnson DM, Wortemann R, McCulloh KA et al A test of the hydraulic vulnerability segmentation hypothesis in angiosperm and conifer tree species. Tree Physiol 2016;36:983–93. 10.1093/treephys/tpw031 27146334

[plaf072-B31] Kaack L, Altaner CM, Carmesin C et al Function and three-dimensional structure of intervessel pit membranes in angiosperms: a review. IAWA J 2019;40:673–702. 10.1163/22941932-40190259

[plaf072-B32] Kaack L, Weber M, Isasa E et al Pore constrictions in intervessel pit membranes provide a mechanistic explanation for xylem embolism resistance in angiosperms. New Phytol 2021;230:1829–43. 10.1111/nph.17282 33595117

[plaf072-B33] Klepsch M, Zhang Y, Kotowska MM et al Is xylem of angiosperm leaves less resistant to embolism than branches? Insights from microCT, hydraulics, and anatomy. J Exp Bot 2018;69:5611–23. 10.1093/jxb/ery321 30184113 PMC6255699

[plaf072-B34] Koçillari L, Olson ME, Suweis S et al The Widened Pipe Model of plant hydraulic evolution. Proc Natl Acad Sci U S A 2021;118:1–8. https://www.pnas.org/doi/full/10.1073/pnas.2100314118 10.1073/pnas.2100314118 PMC8179198 34039710

[plaf072-B35] Kolb KJ, Sperry JS. Differences in drought adaptation between subspecies of sagebrush (*Artemisia tridentata*). Ecology 1999;80:2373–84. 10.1890/0012-9658(1999)080[2373:DIDABS]2.0.CO;2

[plaf072-B36] Kotowska MM, Thom R, Zhang Y et al Within-tree variability and sample storage effects of bordered pit membranes in xylem of *Acer pseudoplatanus*. Trees 2020;34:61–71. 10.1007/s00468-019-01897-4

[plaf072-B37] Kozłowski J, Konarzewski M. Is west, brown and enquist’s model of allometric scaling mathematically correct and biologically relevant? Functional Ecology 2004;18:283–289. 10.1111/j.0269-8463.2004.00830.x

[plaf072-B38] Lazzarin M, Crivellaro A, Williams CB et al Tracheid and pit anatomy vary in tandem in a tall *Sequoiadendron giganteum* tree. IAWA J 2016;37:172–85. 10.1163/22941932-20160129

[plaf072-B39] Lechthaler S, Kiorapostolou N, Pitacco A et al The total path length hydraulic resistance according to known anatomical patterns: what is the shape of the root-to-leaf tension gradient along the plant longitudinal axis? J Theor Biol 2020;502:110369. 10.1016/j.jtbi.2020.110369 32526220

[plaf072-B40] Lens F, Gleason SM, Bortolami G et al Functional xylem characteristics associated with drought-induced embolism in angiosperms. New Phytol 2022;236:2019–36. 10.1111/nph.18447 36039697

[plaf072-B41] Lens F, Sperry JS, Christman MA et al Testing hypotheses that link wood anatomy to cavitation resistance and hydraulic conductivity in the genus Acer. New Phytol 2011;190:709–23. 10.1111/j.1469-8137.2010.03518.x 21054413

[plaf072-B42] Levionnois S, Kaack L, Heuret P et al Pit characters determine drought-induced embolism resistance of leaf xylem across 18 Neotropical tree species. Plant Physiol 2022;190:371–86. 10.1093/plphys/kiac223 35567500 PMC9434246

[plaf072-B43] Levionnois S, Ziegler C, Jansen S et al Vulnerability and hydraulic segmentations at the stem–leaf transition: coordination across Neotropical trees. New Phytol 2020;228:512–24. 10.1111/nph.16723 32496575

[plaf072-B44] Liu M, Pan R, Tyree MT. Intra-specific relationship between vessel length and vessel diameter of four species with long-to-short species-average vessel lengths: further validation of the computation algorithm. Trees 2018;32:51–60. 10.1007/s00468-017-1610-y

[plaf072-B45] Loepfe L, Martinez-Vilalta J, Piñol J et al The relevance of xylem network structure for plant hydraulic efficiency and safety. J Theor Biol 2007;247:788–803. 10.1016/j.jtbi.2007.03.036 17509617

[plaf072-B46] Mouillot D, Bellwood DR, Baraloto C et al Rare species support vulnerable functions in high-diversity ecosystems. PLoS Biol 2013;11:e1001569. 10.1371/journal.pbio.1001569 23723735 PMC3665844

[plaf072-B47] Muggeo Vito M.R . Segmented: An R Package to Fit Regression Models With Broken-Line Relationships. R News 2008;8:20–25. https://api.semanticscholar.org/CorpusID:265349128 (15 December 2025, date last accessed).

[plaf072-B48] Olson ME, Anfodillo T, Gleason SM et al Tip-to-base xylem conduit widening as an adaptation: causes, consequences, and empirical priorities. New Phytol 2021;229:1877–93. 10.1111/nph.16961 32984967

[plaf072-B49] Olson ME, Anfodillo T, Rosell JA et al Universal hydraulics of the flowering plants: vessel diameter scales with stem length across angiosperm lineages, habits and climates. Ecol Lett 2014;17:988–97. 10.1111/ele.12302 24847972

[plaf072-B50] Olson ME, Rosell JA. Vessel diameter-stem diameter scaling across woody angiosperms and the ecological causes of xylem vessel diameter variation. New Phytol 2013;197:1204–13. 10.1111/nph.12097 23278439

[plaf072-B51] Olson ME, Soriano D, Rosell JA et al Plant height and hydraulic vulnerability to drought and cold. Proc Natl Acad Sci U S A 2018;115:7551–6. 10.1073/pnas.1721728115 29967148 PMC6055177

[plaf072-B52] Pan R, Geng J, Cai J et al A comparison of two methods for measuring vessel length in woody plants. Plant Cell Environ 2015;38:2519–26. 10.1111/pce.12566 26084355

[plaf072-B53] Peng G, Geng H, Li Y et al The theory behind vessel length determination using gas flow rates and comparison between two pneumatic methods based on seven woody species. J Exp Bot 2022;73:5612–24. 10.1093/jxb/erac206 35552690

[plaf072-B54] Pereira L, Kaack L, Guan X et al Angiosperms follow a convex trade-off to optimize hydraulic safety and efficiency. New Phytol 2023;240:1788–801. 10.1111/nph.19253 37691289

[plaf072-B55] Pereira L, Miranda MT, Pires GS et al A semi-automated method for measuring xylem vessel length distribution. Theor Exp Plant Physiol 2020;32:331–40. 10.1007/s40626-020-00189-4

[plaf072-B56] Peters JMR, Choat B. Out on a limb: testing the hydraulic vulnerability segmentation hypothesis in trees across multiple ecosystems. Plant Cell Environ 2024;48:2162–77. 10.1111/pce.15249 39562846

[plaf072-B57] Petit G . An appreciation of apex-to-base variation in xylem traits will lead to more precise understanding of xylem phenotypic plasticity. New Phytol 2024;244:1175–80. 10.1111/nph.20109 39262308

[plaf072-B58] Petit G, Anfodillo T. Plant physiology in theory and practice: an analysis of the WBE model for vascular plants. J Theor Biol 2009;259:1–4. 10.1016/j.jtbi.2009.03.007 19289132

[plaf072-B59] Petit G, Anfodillo T, Mencuccini M. Tapering of xylem conduits and hydraulic limitations in sycamore (*Acer pseudoplatanus*) trees. New Phytol 2008;177:653–64. 10.1111/j.1469-8137.2007.02291.x 18069964

[plaf072-B60] Petit G, Mencuccini M, Carrer M et al Axial conduit widening, tree height, and height growth rate set the hydraulic transition of sapwood into heartwood. J Exp Bot 2023;74:5072–87. 10.1093/jxb/erad227 37352139

[plaf072-B61] Pittermann J, Sperry JS, Wheeler JK et al Mechanical reinforcement of tracheids compromises the hydraulic efficiency of conifer xylem. Plant Cell Environ 2006;29:1618–28. 10.1111/j.1365-3040.2006.01539.x 16898022

[plaf072-B62] Plavcová L, Jansen S, Klepsch M et al Nobody’s perfect: can irregularities in pit structure influence vulnerability to cavitation? Front Plant Sci 2013;4:453. 10.3389/fpls.2013.00453 24273549 PMC3824106

[plaf072-B63] Pourahmadazar J, Ghobadi C, Nourinia J. Novel modified pythagorean tree fractal monopole antennas for UWB applications. IEEE Antennas Wirel Propag Lett 2011;10:484–7. 10.1109/LAWP.2011.2154354

[plaf072-B64] Price CA, Enquist BJ. Scaling of mass and morphology in plants with minimal branching: an extension of the WBE model. Funct Ecol 2006;20:11–20. 10.1111/j.1365-2435.2006.01078.x 17536400

[plaf072-B65] R Core Team . R: A Language and Environment for Statistical Computing. Vienna, Austria: R Foundation for Statistical Computing 2020.

[plaf072-B66] Rosell JA, Olson ME, Anfodillo T. Scaling of xylem vessel diameter with plant size: causes, predictions, and outstanding questions. Curr For Rep 2017;3:46–59. 10.1007/s40725-017-0049-0

[plaf072-B67] Schindelin J, Arganda-Carreras I, Frise E et al Fiji: an open-source platform for biological-image analysis. Nat Methods 2012;9:676–82. 10.1038/nmeth.2019 22743772 PMC3855844

[plaf072-B68] Schmid R, Machado RD. Pit membranes in hardwoods-fine structure and development I. Protoplasma 1968;66:185–204. 10.1007/BF01252532

[plaf072-B69] Scholz A, Klepsch M, Karimi Z et al How to quantify conduits in wood? Front Plant Sci 2013;4:56. 10.3389/fpls.2013.00056 23507674 PMC3600434

[plaf072-B70] Sorek Y, Greenstein S, Netzer Y et al An increase in xylem embolism resistance of grapevine leaves during the growing season is coordinated with stomatal regulation, turgor loss point and intervessel pit membranes. New Phytol 2021;229:1955–69. 10.1111/nph.17025 33098088

[plaf072-B71] Soriano D, Echeverría A, Anfodillo T et al Hydraulic traits vary as the result of tip-to-base conduit widening in vascular plants. J Exp Bot 2020;71:4232–42. 10.1093/jxb/eraa157 32219309

[plaf072-B72] Sperry JS, Hacke UG, Pittermann J. Size and function in conifer tracheids and angiosperm vessels. Am J Bot 2006;93:1490–500. 10.3732/ajb.93.10.1490 21642096

[plaf072-B73] Sperry JS, Hacke UG, Wheeler JK. Comparative analysis of end wall resistivity in xylem conduits. Plant Cell Environ 2005;28:456–65. 10.1111/j.1365-3040.2005.01287.x

[plaf072-B74] Tyree MT, Ewers FW. The hydraulic architecture of trees and other woody plants. New Phytol 1991;119:345–60. 10.1111/j.1469-8137.1991.tb00035.x

[plaf072-B75] Tyree MT, Zimmermann MH. Xylem Structure and the Ascent of Sap, 2nd edn. Berlin, Germany: Springer Series in Wood Science, 2002, 28–201.

[plaf072-B76] West GB, Brown JH, Enquist BJ. A general model for the origin of allometric scaling laws in biology. Science 1997;276:122–6. 10.1126/science.276.5309.122 9082983

[plaf072-B77] West GB, Brown JH, Enquist BJ. A general model for the structure and allometry of plant vascular systems. Nature 1999;400:664–7. 10.1038/23251

[plaf072-B78] Wheeler JK, Sperry JS, Hacke UG et al Inter-vessel pitting and cavitation in woody Rosaceae and other vessel led plants: a basis for a safety versus efficiency trade-off in xylem transport. Plant Cell Environ 2005;28:800–12. 10.1111/j.1365-3040.2005.01330.x

[plaf072-B79] Wickham Hadley . ggplot2: Elegant Graphics for Data Analysis, 2nd edn. Berlin, Germany: Springer, 2016, 241–53. 10.1007/978-3-319-24277-4

[plaf072-B80] Williams CB, Anfodillo T, Crivellaro A et al Axial variation of xylem conduits in the Earth’s tallest trees. Trees 2019;33:1299–311. 10.1007/s00468-019-01859-w

[plaf072-B81] Zach A, Schuldt B, Brix S et al Vessel diameter and xylem hydraulic conductivity increase with tree height in tropical rainforest trees in Sulawesi, Indonesia. Flora Morphol Distrib Funct Ecol Plants 2010;205:506–12. 10.1016/j.flora.2009.12.008

[plaf072-B82] Zambonini D, Savi T, Rosner S et al Consistent decrease in conifer embolism resistance from the stem apex to base resulting from axial trends in tracheid and pit traits. Front Plant Sci 2024;15:1414448. 10.3389/fpls.2024.1414448 38988629 PMC11234846

[plaf072-B83] Zanne AE, Sweeney K, Sharma M et al Patterns and consequences of differential vascular sectoriality in 18 temperate tree and shrub species. Funct Ecol 2006;20:200–6. 10.1111/j.1365-2435.2006.01101.x

[plaf072-B84] Zhang Y, Pereira L, Kaack L et al Gold perfusion experiments support the multi-layered, mesoporous nature of intervessel pit membranes in angiosperm xylem. New Phytol 2024;242:493–506. 10.1111/nph.19608 38404029

[plaf072-B85] Zimmermann MH, Brown CL. Trees—Structure and Function. Berlin, Germany: Springer, 1971.

[plaf072-B86] Zimmermann MH, Jeje AA, Jeje AA. Vessel-length distribution in stems of some American woody plants. Can J Bot 1981;59:1882–92. 10.1139/b81-248

[plaf072-B87] Zimmermann MH, Tomlinson PB. Analysis of complex vascular systems in plants: optical shuttle method. Science 1966;152:72–3. 10.1126/science.152.3718.72 17830235

