# Supplementary material for: Xylem vessel anatomy and hydraulic function scale in concert along the tip-to-base axis of an angiosperm tree
Source: AoB Plants. 2025 Dec 10;17(6):plaf072. doi: 10.1093/aobpla/plaf072 (PMC12741662; doi:10.1093/aobpla/plaf072)
Supplement: plaf072_Supplementary_Data [file plaf072_supplementary_data.zip › Supporting Information.pdf]

Supporting Information

We identified a linear relationship of vessel diameter, as well as mean vessel length from tip to base of the stem, as well as the first and second order branches.

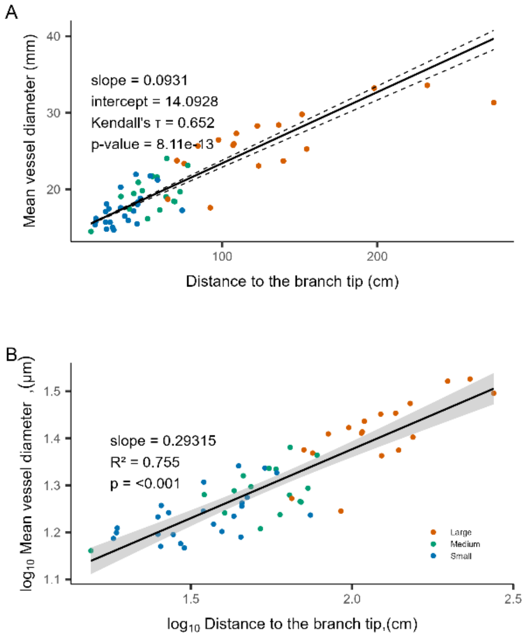

Figure S 1 Relationship between the mean vessel diameter (µm) (A) and log-transformed vessel diameter (B) and segment position in the tree separated by the segment diameter sampled. The black line indicates the non-parametric linear regression of the pooled data (n = 58) with 95% CI (dashed lines). Each dot represents a value for a segment of the sapling studied.

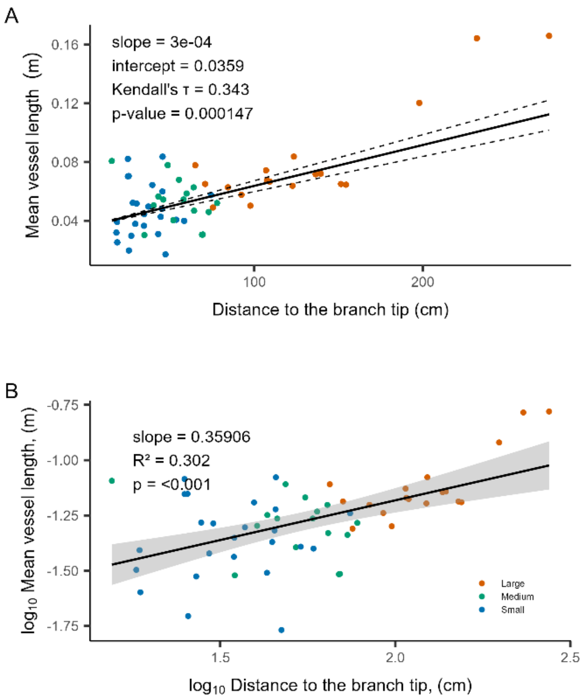

Figure S 2 Relationship between the mean vessel length (m) (A) and log-transformed vessel diameter (B) and segment position in the tree separated by the segment diameter sampled. The black line indicates the non-parametric linear regression of the pooled data (n = 58) with 95% CI (dashed lines).

We identified a weak relationship between axial height and intervessel contact fraction (F_C), as well as pit field fraction (F_{PF}), indicating low or no scaling.

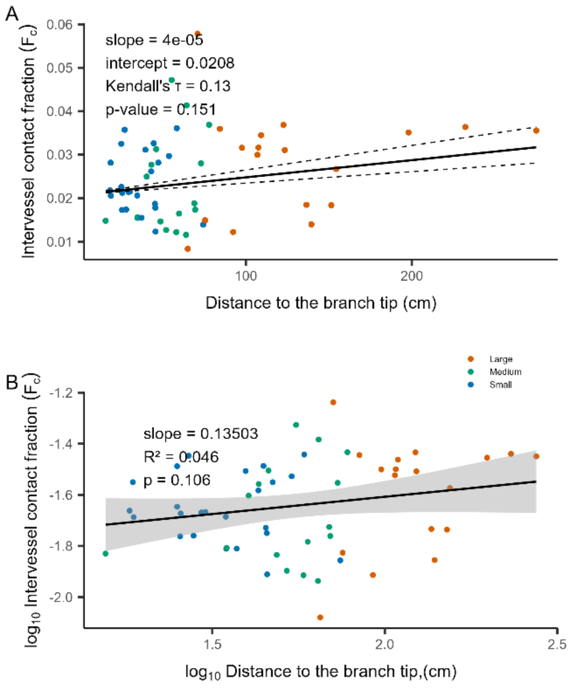

Figure S 3 Relationship between the intervessel contact fraction (unitless) with a non-parametric linear regression (A) and log- transformed data (B) and segment position in the tree separated by segment diameter. Both parameters show weak scaling across the tree height (n = 58).

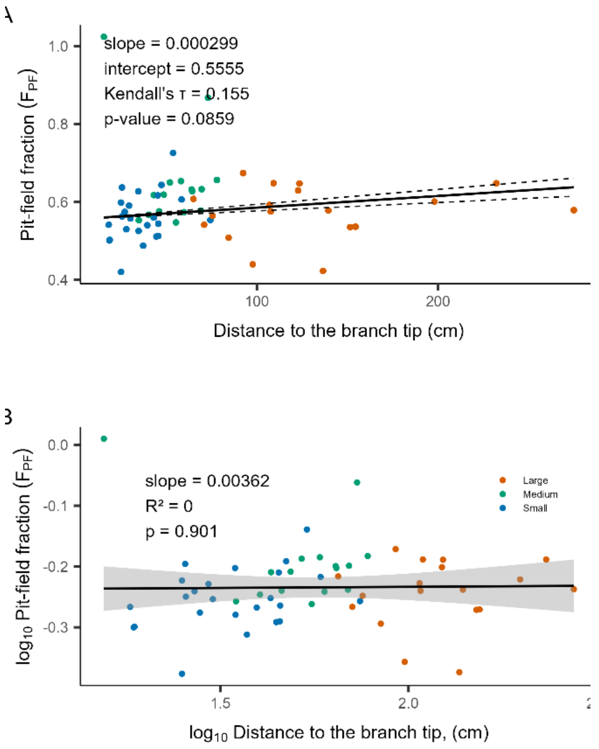

S

Figure S 4 Relationship between the pit- field fraction (unitless) and segment position in the tree separated by segment diameter with a non-parametric linear regression (A) and log-transformed data (B). Both parameters show weak scaling across the tree height (n = 58).

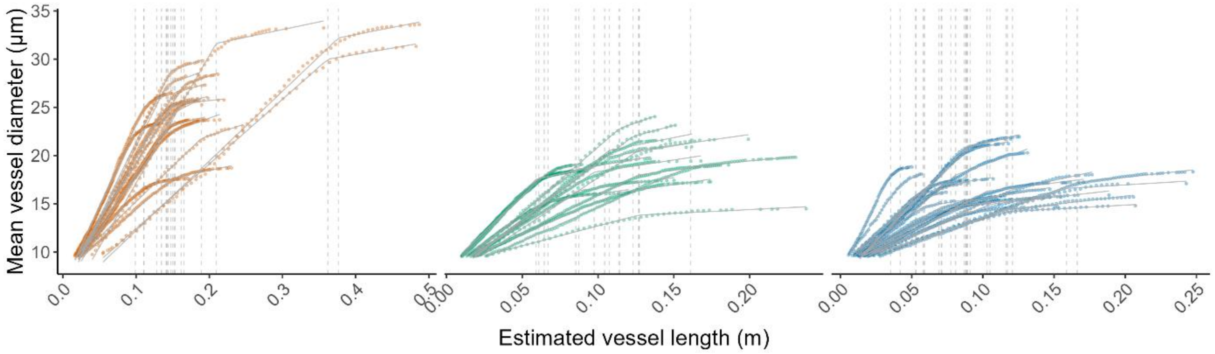

Figure S 5 Measured vessel diameter in relation to the estimated, grouped vessel length for each segment, each indicated by a sperate colour. Each point represents a bin of vessels (n = 10) with a measured diameter. The grey dashed line represents the piecewise linear regression for each sample grouped by large segment diameter (orange), medium segment diameter (green) and small segment diameter (blue). The vertical dashed lines represent the break points of the piecewise linear regression. (See the material and methods section: Relating classes of vessel length to corresponding vessel diameter classes).

Branches of the second order had a smaller segment diameter. The decreasing slope in Figure S 5 indicated that an increase of the estimated vessel length was subtler for increasing vessel diameters, and, in general, the vessels were longer compared to those with a similar diameter from the main stem. The plateau when reaching a maximum mean vessel diameter depended on the estimated vessel length: shorter vessels reached this plateau generally at narrower vessel diameters, especially for samples from the main stem. The vessels followed a skewed distribution pattern, with most vessels being narrow and short, then reaching a point where the diameter was increasing only slightly with increasing vessel length.

Our estimations of the pressure difference across vessel end walls showed a convex relationship with vessel end wall conductivity within the sapling studied (Figure S 6). High pressure differences across a vessel end wall were associated with low values of A_P , which could be found at the tips of the branches or the tip of the main stem (Figure S 6).

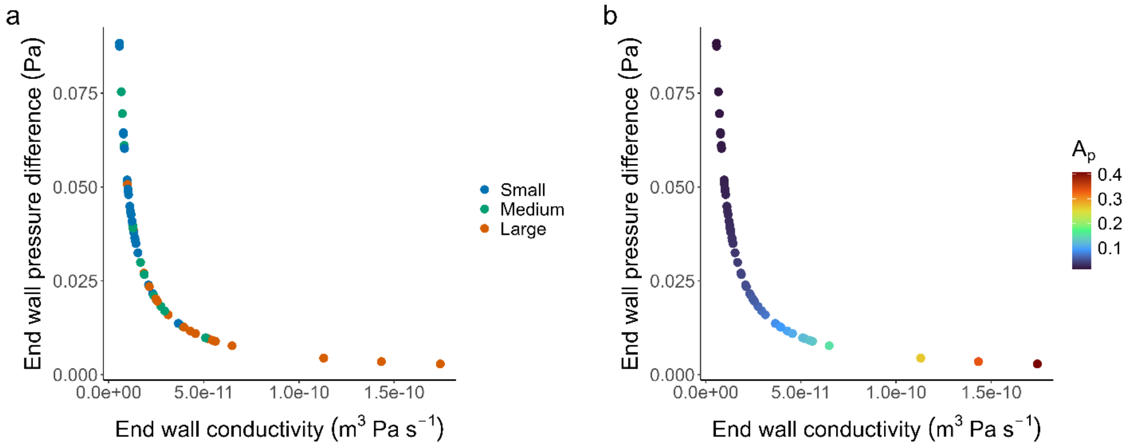

Figure S 6 Vessel end wall conductivity in relation to the pressure difference generated across an intervessel pit field for various segments of a *Fagus sylvatica* sapling (**a**). Variation in the total intervessel pit membrane area per vessel (A_P; mm^2) is shown in **b**.

The convex relationship within a single tree highlighted the adjustment of the large diameter segments (orange) with respect to hydraulic conductivity, while intermediate segment diameters (green) and the tips of branches (small segment diameters in blue) were found at the resistant end of the convex pattern.

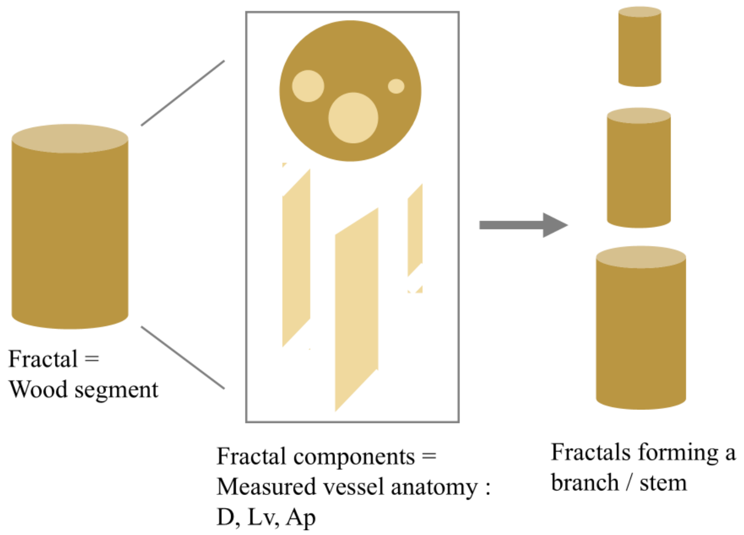

Figure S 7 Conceptual illustration of a wood segment interpreted as a fractal unit. Each segment (fractal) contains vessels characterized by specific anatomical traits such as vessel diameter (D) with the according vessel length (Lv) (Figure S5), as well as A_P and T_{PM}. These components collectively define the fractal structure. Several of these segments can be assembled to represent the hierarchical organization of a branch or stem, highlighting the scaling relationships across a branch (Figure S 1 & Figure S2).

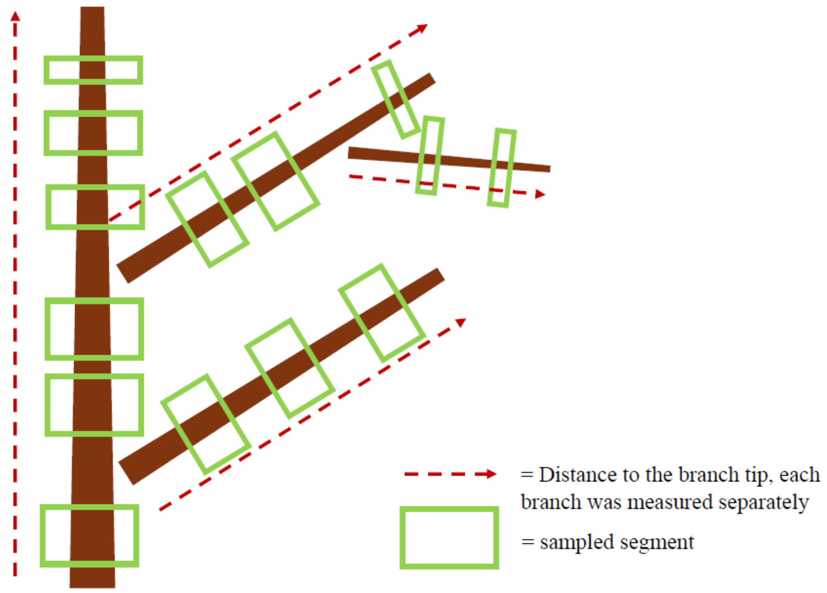

Figure S 8 Sampling scheme. Each Branch length was measured (distance to the branch tip) and divided in segments (green) that were used to obtain anatomical data.
